# Identification of Peste des Petits Ruminants Transmission Hotspots in the Karamoja Subregion of Uganda for Targeting of Eradication Interventions

**DOI:** 10.3389/fvets.2019.00221

**Published:** 2019-07-05

**Authors:** Joseph Nkamwesiga, Jeanne Coffin-Schmitt, Sylvester Ochwo, Frank Norbert Mwiine, Annabella Palopoli, Christian Ndekezi, Emmanuel Isingoma, Noelina Nantima, Peninah Nsamba, Rogers Adiba, Saskia Hendrickx, Jeffrey C. Mariner

**Affiliations:** ^1^College of Veterinary Medicine, Animal Resources and Biosecurity, Makerere University, Kampala, Uganda; ^2^Cummings School of Veterinary Medicine, Tufts University, Grafton, MA, United States; ^3^Department of Animal Health, Ministry of Agriculture, Animal Industry and Fisheries, Entebbe, Uganda; ^4^Mercy Corps, Kampala, Uganda; ^5^Feed the Future Innovation Lab for Livestock Systems, University of Florida, Gainesville, FL, United States

**Keywords:** Peste des petits ruminants, Uganda, participatory epidemiology, eradication, Karamoja

## Abstract

This paper describes an assessment of the patterns of *peste des petits ruminants* virus circulation in the Karamoja subregion of Uganda conducted to identify the communities that maintain the virus and inform the development of a targeted vaccination strategy. Participatory epidemiological methods were used to develop an operational hypothesis for the patterns of PPR in Karamoja that was subsequently validated through outbreak investigation and genomics. The participatory epidemiological assessment included risk mapping with livestock owners, community animal health workers and veterinarians and indicated there were two critical foci of virus transmission on the Uganda-Kenya border. One was located in two adjacent subcounties of Kotido and Kaabong Districts in northern Karamoja and the other in Loroo subcounty of Amudat District in southern Karamoja. Participants reported that these were locations where outbreaks were usually first observed in Karamoja and subsequently spread to other areas. Following the participatory assessment, surveillance activities were implemented across the Karamoja subregion in 2018. Three outbreak were detected, investigated and sampled. Two outbreaks were located in the northern and one on the southern focus of transmission. No Outbreaks were diagnosed in Karamoja outside of these foci during 2018. Genomics indicated different clusters of viruses were associated with the northern and southern foci that were more closely related to other East African isolates than to each other. This indicates these are two separate systems of virus circulation which should be explicitly addressed in eradication as separate cross-border systems that require integrated cross-border interventions.

## Introduction

*Peste des petits ruminants* (PPR) is a highly contagious and fatal disease of sheep and goats that negatively impacts the livelihoods, and food and nutritional security of livestock farmers throughout large parts of Africa, Asia, and the Middle East (1–4). Small ruminants play a central role in household economies as ready sources of cash and protein, especially for women and children. PPR virus is closely related to rinderpest (RP), the first livestock disease to be globally eradicated. Effective vaccines, including thermostable vaccines, are available for the control of PPR (5). After the successful global eradication of RP, the international community identified PPR, the closest relative of RP, for global eradication by 2030 (6).

The primary challenges to the eradication of PPR are the large size of small ruminant populations and their short life span. These demographic concerns create the need to target vaccination to critical points in viral maintenance systems using fit-to-purpose, public-private-community partnerships that effectively harness incentives for participation to interrupt disease transmission (7).

This paper describes an assessment of the patterns of PPR virus (PPRV) circulation in the Karamoja subregion of Uganda to develop a vaccination strategy targeted to the rural pastoral communities responsible for maintaining the virus. Karamoja is a pastoral region of Uganda bordering South Sudan and Kenya principally. This region is predominantly occupied by Karamojong and Pokot peoples practicing transhumance where cattle are kept in mobile camps often referred to in English as kraals. The Karamojong cluster of tribes speak related dialects and includes three communities in Uganda (Dodoth, Jie, and Karamojong), one in Kenya (Turkana) and the two in South Sudan (Toposa and Jie). The Pokot speak a Kalenjin language and occupy southern Karamoja residing on both sides of the Kenya-Uganda border. A combination of participatory and laboratory-based epidemiological methods were used in sequence to develop and then test an operational hypothesis for understanding the endemic patterns of PPR in Karamoja.

## Materials and Methods

The action research combined participatory epidemiological field assessments with participatory risk mapping exercises to develop qualitative risk maps. The surveillance system was then reinforced across Karamoja to actively search, sample and diagnose outbreaks of PPR using participatory methods. Finally, serology and genetic analysis was conducted on materials collected during investigations to test the epidemiological scenario developed during the participatory phase of the assessment.

### Site Assessment and Risk Mapping

**Figure 1:**
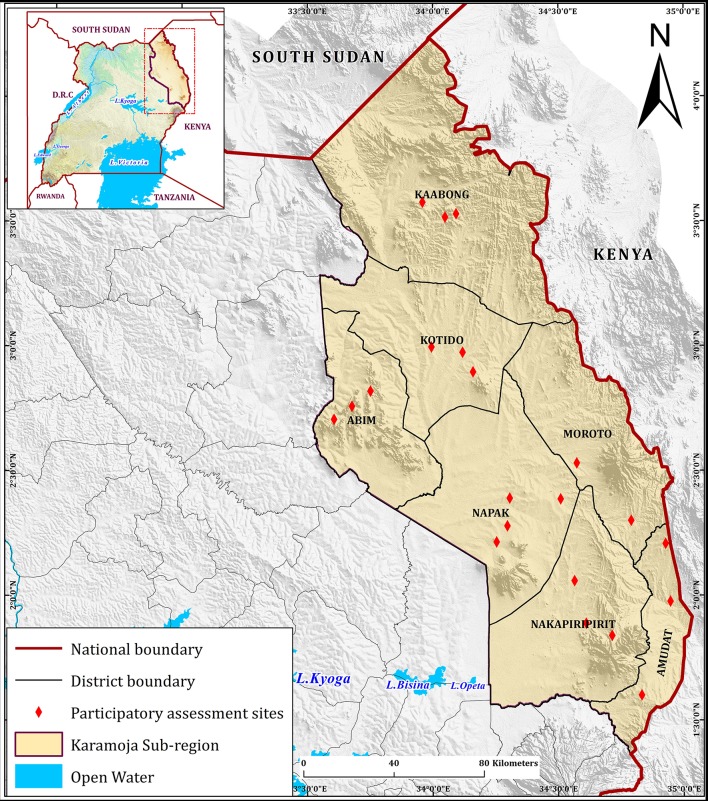
Karamoja participatory assessment sites. Participatory assessment sites were distributed throughout all districts of Karamoja.

A site assessment of the Karamoja sub-region was undertaken to assess the patterns of PPR transmission in the area and identify transmission hotspots for targeting of control interventions using participatory epidemiology. The site assessment studied the animal health situation, community animal health knowledge, and the quality and availability of animal health services in the region. Semi-structured interviews with focus groups and key informants were conducted in all the seven districts of Karamoja as defined at the time of project design (Figure 1). Karamoja was redistricted over the course of project implementation and study results are presented using current maps at the preference of respondents. A minimum of three focus groups with kraal (livestock camp) leaders and livestock owners, and three key informant interviews with veterinary drug shop owners were conducted in each of the seven districts. Other key informants included governmental extension officials, district veterinary personnel, and animal health workers. The project team took the opportunity to conduct two interviews with Turkana herders present in Kaabong. Twenty-one focus groups, 14 veterinary drug shop interviews, and 9 governmental interviews were held. Semi-structured interviews with livestock owners and drug shop owner/operators followed an internal review board approved outline tailored to their livelihood. Mapping, proportional piling, and timelines were the main visualization techniques used during interviews (8). Data were collected in notes and pictures, and key themes were extracted. These data were combined and cross-checked with information on PPR disease, livelihoods, and animal health services from business model and epidemiology training workshops with community animal health workers, veterinary officials, and veterinary drug shop owners.

Risk maps were prepared in two meetings that included veterinarians, community animal health workers and kraal leaders from all districts of Karamoja. Participants were organized into focus groups according to their home areas and asked to produce local risk maps for PPR by listing the risk factors for PPR and then indicating the distribution of each category of risk on a poster paper. Groups presented their maps to the meeting and a discussion followed. The participants were then reorganized into two mixed groups with representation from all districts and asked to prepare a new risk map synthesizing all of Karamoja. Subsequently, these same groups drew timelines representing the epidemic curves of the annual incidence of PPR from the time of their first observation of PPR.

The project then defined target sites for vaccination interventions based on the risk maps, transmission foci indicated and available resources for vaccination.

### Surveillance

Surveillance for PPR was supported through training in participatory surveillance (9, 10) and back-stopping outbreak investigation via an agreed protocol for integrated project partner response. Participatory surveillance is a sensitive technique for finding disease outbreaks using syndromic case definitions that are consistent with the target disease and subsequently confirming their diagnosis with biological tests. In the case of PPR, a suspect outbreak was defined as any clinical event that met a stomatitis-enteritis syndromic case definition. Suspect outbreaks were then confirmed with PPR rapid field tests and PCR.

Fourteen locally active individuals participated in a 10-day training on PS for PPR, including two non-governmental organization workers, two Makerere graduate students, and 10 governmental veterinary officials from each of the seven Karamoja districts. One kit with appropriate materials for a PPR outbreak investigation was provided to each district veterinary office. Attendees were instructed in their roles in the outbreak investigation and response protocol. This protocol for outbreak investigation was agreed upon among project partners: Ministry of Agriculture, Animal Industries and Fisheries (MAAIF)-Uganda, Tufts University's Cummings School of Veterinary Medicine (Tufts), Makerere University (MaK), Mercy Corps Uganda, and the Karamoja Veterinary Laboratory (KVL). Communication with official channels was key to enable governmental officials to promptly respond to any new outbreaks. It included the use of interviews, rapid field tests (PESTE-TEST, Pirbright Inst) (11), and PCR tests to confirm suspected outbreaks.

Sampling at outbreaks included ocular and nasal swabs, serum and whole blood. All samples were stored on ice and delivered within 48 h to the Molecular Biology Laboratory, College of Veterinary Medicine, Animal Resources and Biosecurity, Makerere University.

### Serology

Four sets of serological samples were collected. Each of the two hotspots identified in the participatory risk-mapping and the immediate areas of the Loroo and Kamion outbreak sites at the time of the outbreak were sampled. Sampling sites were selected using randomly generated geographic coordinates within the targeted communities; the nearest herd to the coordinates was selected. Sample size was estimated using a 70% estimate of prevalence, an error of ±4%, and a design effect of 1.2 to account for within herd clustering effects. In the two hot spots sampled, 25 sites in each of two communities were selected. A total of 28 animals, or all animal present in smaller herds, were sampled in each herd using a systematic sampling method. In the outbreak samples, 20 and 22 herds were selected in Loroo and Kamion, respectively. A total of 700 small ruminants were sampled for each of the two hotspots for serosurvey whereas 478 and 440 small ruminants were sampled during the Loroo Subcounty and Kamion Subcounty outbreak investigations, respectively.

Peste des Petits Ruminants virus specific antibodies in sera were tested using a commercial competitive ELISA platform, ID Screen® PPR Competition [ID_Vet Grabels, France]. The assay was performed following the manufacture's OIE-recommended protocol (12). The cutoffs were calculated as $\frac{S}{N}\left(\%\right)=\frac{OD_{sample}}{OD_{Negative\;contrrol}}*100$.

The samples with percentage inhibition (S/N) ≤60% were considered positive whereas samples with (S/N) value above 60% were negative.

### RNA Extraction From Swabs

RNA was extracted using a commercial RNA extraction kit (Zymo Research, USA). Two hundred and fifty microliter of each swab sample was homogenized with 500 μl of Trizol reagent™ with a vortex mixer for 30 s. Five hundred microliter of absolute ethanol was added to the sample homogenate and transferred into a Zymo-Spin™ III CG Column and centrifuged at 10,000 × g for 30 s until all was finished. Eighty microliter of DNAse 1 solution was added to each column and incubated for 15 min at room temperature before washing with 400 μl of Direct-zol™ RNA PreWash™ buffer. The column was then washed with 700 μl of RNA Wash Buffer and centrifuged for 2 min to ensure complete removal of the wash buffer. RNA was eluted in 50 μl of nuclease-free water. Extracted RNA was either immediately used for cDNA synthesis or kept at −80°C until required.

### Copy DNA (cDNA) Synthesis

LunaScript® RT SuperMix Kit (New England Biolabs, USA) was used for cDNA synthesis. cDNA was prepared in a 20 μl reaction containing 4 μl of LunaScript® RT SuperMix (5X), 5 μl of extracted RNA and 11 μl of Nuclease-free water. The PCR tubes were then placed into a thermocycler for 1 cycle of primer annealing of 25° C for 2 min, cDNA synthesis at 55° C for 10 min and heat inactivation at 95°C for 1 min. The synthesized cDNA was immediately used for PCR or stored at −20° C.

### F and N Gene PCR Amplification

Polymerase chain reaction (PCR) was performed on the synthesized cDNA with two pairs of primers PPRVF1b: [ 5′AGTACAAAAGATTGCTGATCACAGT 3′]

PPRVF2d: 5′GGGTCTCGAAGGCTAGGCCCGAATA 3′ and NP3 (5′-TCTCGGAAATCGCCTCACAGACTG-3′) and NP4 (5′-CCTCCTCCTGGTCCTCCAG AATCT-3′) which target 448 and 351 bp fragments, respectively, as previously described (13, 14).

The PCR reaction was performed in a 20 μl reaction containing 10 μl Taq DNA Pol 2.0X MyTaqRedMix (Bioline, UK), 1 μl (10 μM) of each primer, 3 μl of cDNA and 5 μl of nuclease-free water (Qiagen, USA). The mixture was then subjected to an initial denaturation at 95°C for 5 min followed by 35 cycles of denaturation at 94°C for 30 s, annealing at 55°C for 30 s and extension at 72°C for 2 min and final extension at 72°C for 7 min. Amplification was performed in a S1000 ™ Thermal Cycler [BIO RAD, California, United states]. Ten microliter of each PCR amplicon were resolved on a 2% ethidium bromide-stained agarose gel as previously described (14–16).

### Nucleotide Sequencing

Purified PCR products were shipped to INQABA BIOTEC (Pretoria, South Africa) and sequenced using the ABI 3500XL Genetic Analyzer, POP7™, BrilliantDye™ Terminator v3.1 [Thermo Fisher Scientific, USA]. The nucleotide sequences were deposited in the GenBank under accession numbers MK250004-MK2500011 and MK242028-MK2242037 for the Nucleoprotein and Fusion genes, respectively.

### Spatial Analysis

ELISA result data and GPS coordinates were entered in Microsoft excel office 2016 package and saved as coma separated files [.csv]. Seroprevalence data was used to map the spatial distribution of PPR antibodies in the foci in the Karamoja subregion. The shape files were obtained from the web-based GIS free resource while some were created in ArcMap ver. 10.5 software. Point prevalence data from the two foci were interpolated using an inverse-distance weighted (IDW) technique using the Geostatistical Analyst tool in ArcMap ver. 10.5.

### Data Analysis

Triangulation of information, as practiced in participatory rural appraisal (2, 8) was utilized to compare results within the participatory assessments and with epidemiological data such as historical vaccination coverage. Triangulation was also used between the participatory assessments and laboratory-based test results.

In preparation for statistical analysis, data was entered into Microsoft Excel (Office 2016 Package) for curation. Overall seroprevalence was calculated by dividing the number of positive animals by the total number of small ruminants sampled whereas the herd level prevalence was calculated by dividing the number of positive animals the number of animals each herd (village) contributed.

The raw nucleotide sequences (ab1 files) were viewed and edited with BioEdit software version 7.0.0 (17) to remove any ambiguous sequences. Multiple sequence alignments and phylogenetic trees were constructed using MEGA X software (18, 19). The representative sequences for each lineage with which to compare were downloaded from the web-based GenBank housed at the NCBI for each gene sequence.

## Results

### Site Assessment and Risk Mapping

The site assessment interviews indicated that PPR was a common and important problem. The majority of communities were able to recall and describe outbreaks of disease consistent with PPR. The names used by the Karamoja for the disease, 14 recorded in total, were diverse and localized whereas the Pokot tended to use a single name, *losür*. Not all respondents who could describe syndromes and events consistent with PPR used a name specific for the disease; some used a name that could describe multiple diseases. Commonly, participants stated the disease outbreaks were introduced to Karamoja region from Kenya and then move west deeper into Uganda as the communities practiced seasonal movements in search of grazing. No reports of outbreaks arising from the West were received.

**Figure 2:**
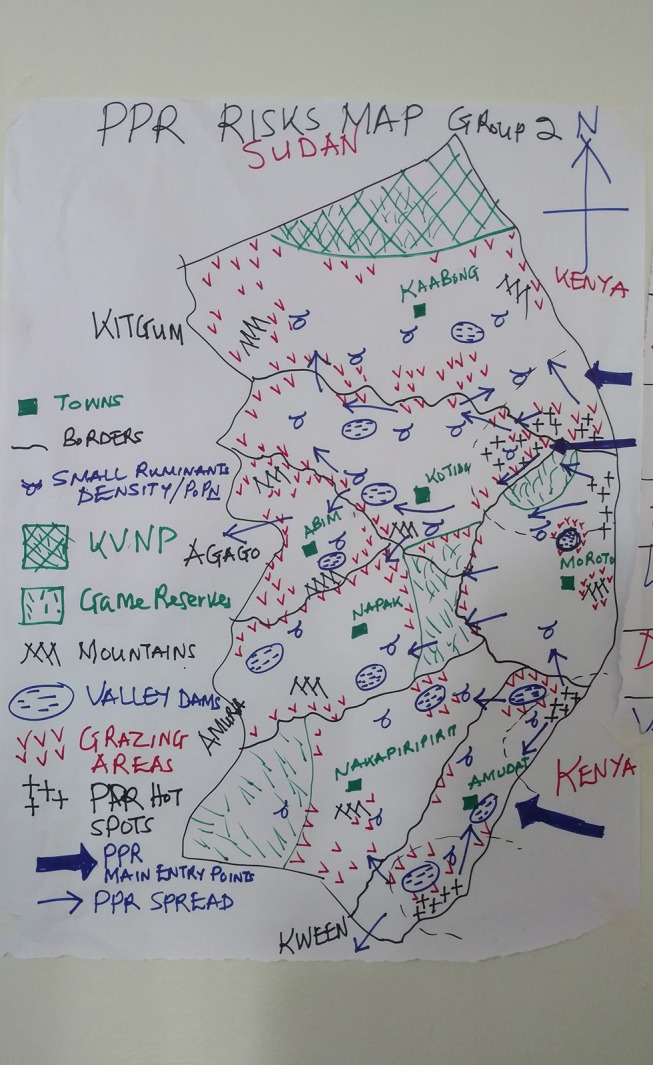
Example of a Karamoja Risk Map. Example of a risk map drawn by a focus group of veterinarians, community animal health workers and kraal leaders from across Karamoja. The thick blue arrows indicate the perceived main entry points for PPR to Karamoja and the thin blue arrows represent spread across Karamoja. Black crosses are PPR transmission hotspots. The northern focus includes Nakapelimoru subcounty of Kotido, Loyoro subcounty of Kaabong District. The southern focus is centered on Loroo sub county of Amudat. A third focus is indicated in the extreme south in Karita sub county of Amudat was not included in the project for financial reasons.

Hot spots for PPR were delineated based on the triangulated site assessment data and the risk maps prepared by the focus groups (Figure 2). The map indicates that there are two principle hot-spots for PPR. The southern focus is centered on the border area between Loroo Subcounty of Amudat District, Uganda and the Alalae area of West Pokot District in Kenya. The local Pokot community reported that they were fully integrated across the border. They and their livestock moved freely between Uganda and Kenya. Many respondents used Kenyan cell phone numbers and Kenya currency was commonly used in the area.

The northern focus is at the intersection of Kotido, Kaabong, and Moroto Districts. It includes Nakapelimoru subcounty in Kotido District, Loyoro subcounty in Kaabong District of Uganda. The adjacent Kobebe dam area in the north of Moroto District was also mentioned in the focus groups and site assessments interviews as a component of the northern focus of transmission. Turkana from the Loima area of Kenya frequently share the grazing in the northern hotspot and reported livestock disease profiles that were consistent with the information provided by Karamojong participants.

In the focus groups, there was a broad consensus the first PPR compatible events in Karamoja in modern memory date from 2005. From the time of the first recognition of PPR by local communities in 2005 until present, the direction of virus flow was reported to be from the East to West (Figure 2).

**Figure 3:**
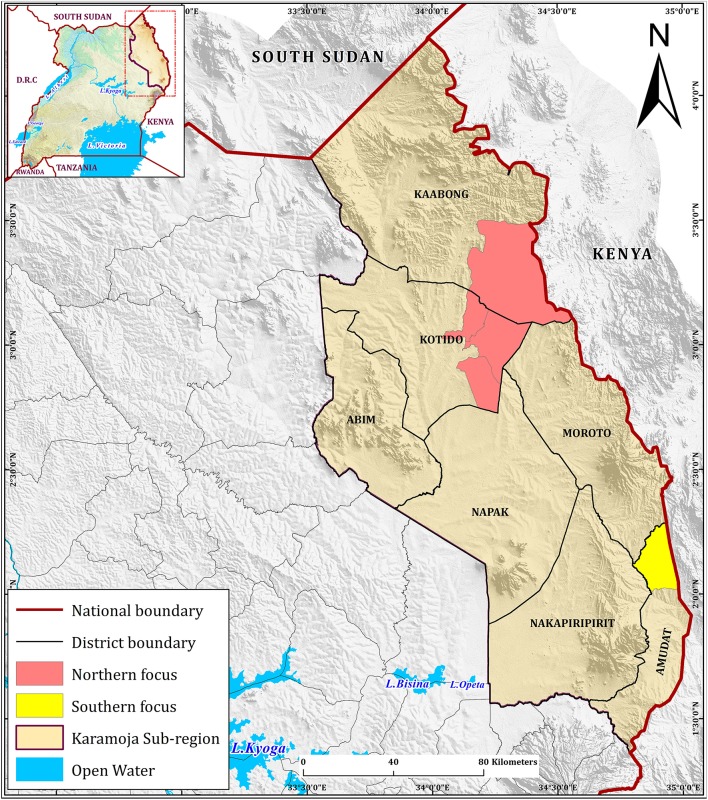
Target sites. The foci identified by the epidemiological assessment are highlighted in red and yellow. The three outbreaks identified by the regional surveillance activity fell within the two foci: two in the northern foci and one in the southern focus.

The project then identified target areas in Uganda for the focus of control interventions along the border with Kenya (Figure 3). One hypothesized critical hotspot in the north is shown and includes Nakapelimoru Subcounty in Kotido District and Loyoro Subcounty in Kaabong District of Uganda. The second was to the south in Loroo Subcounty, Amudat district.

### Surveillance

Surveillance identified three outbreaks, all of which were in the two hotspots identified in the participatory assessment and risk mapping. The locations were Loroo Subcounty in Amudat District, Nakapelimoru Subcounty in Kotido District and Kamion Subcounty in Kaabong District. The outbreaks in Loroo and Kamion were positive using the field rapid test. The Nakapelimoru outbreak was not tested with the field rapid test. All three outbreaks were positive on PCR. Herds in the Kamion outbreak were housed together in large defensive kraals due to recent problems with raiding. Herds in Loroo and Nakapelimoru were kept separately at homesteads.

### Serology

**Figure 4:**
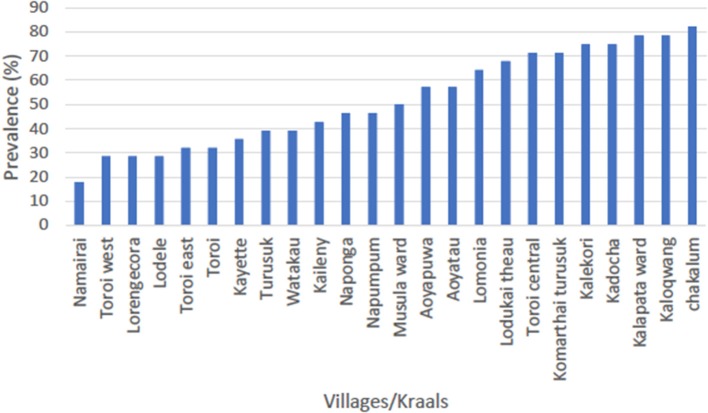
Serosurvey result for the northern focus of transmission (Kotido-Kaabong). The average prevalence in the northern focus was 51.4% with the highest seroprevalence being 82.1% in Chakalum village whereas the lowest was 17.9% in Namairai.

**Figure 5:**
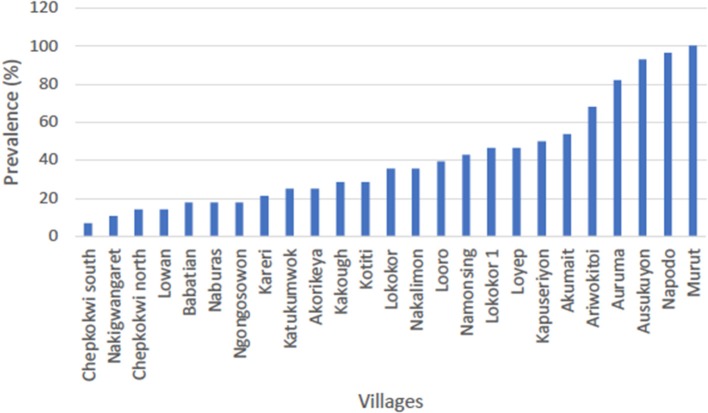
Serosurvey result for the southern focus of transmission (Amudat). The average prevalence in Loroo subcounty was 40.7% with the highest seroprevalence being 100% in Murut village whereas the lowest was 7.1% in Chepkokwi south. More than half of the sampled villages (herds) had prevalence below 50%.

**Figure 6:**
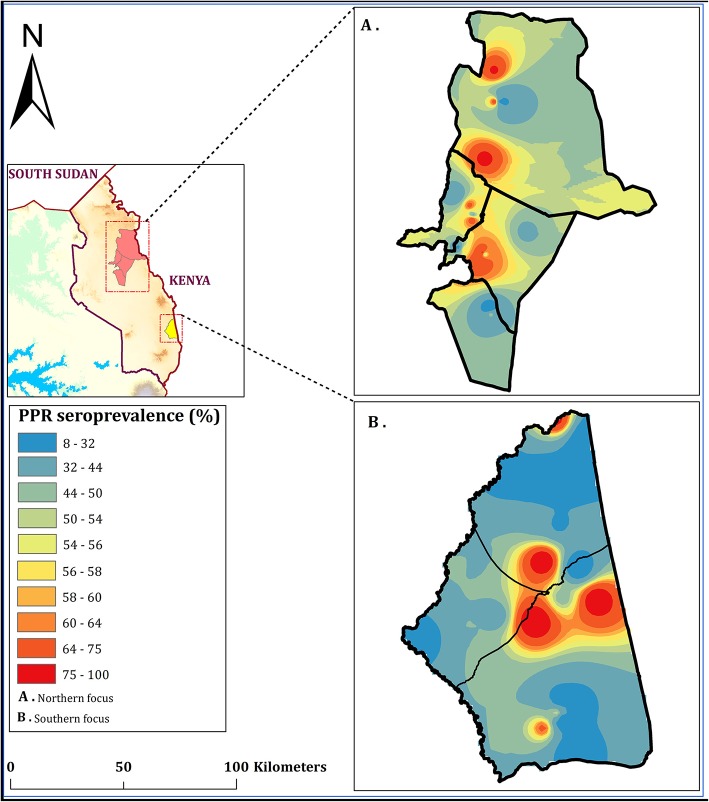
Seroprevalence of PPR in Karamoja subregion (in set); May 2018. Shows the spatial distribution of PPR in the northern focus **(A)** and the southern focus **(B)** interpolated using 25 PPR point prevalence in each focus to create a focus-wide spatial effect. Interpolations were made using inverse-distance weighted (IDW) method using the Geostatistical Analyst tool in ArcMap ver. 10.5 software to produce a continuous *PPR* prevalence raster map on a spectral color ramp.

**Figure 7:**
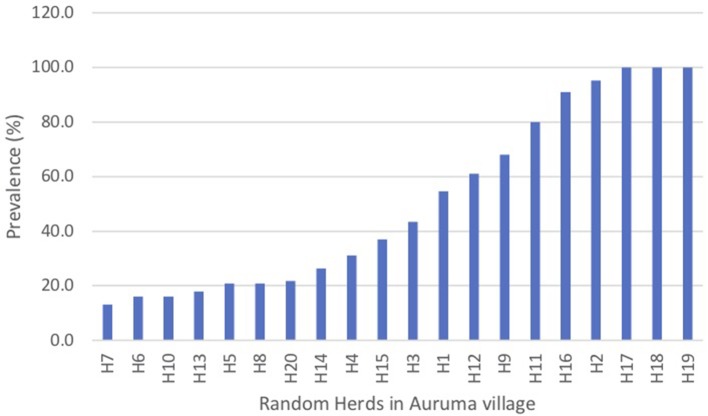
Serosurvey results from the Loroo outbreak investigation in the southern transmission focus (Amudat). It showed the distribution of prevalences was uniformly greater in comparison with the baseline serosurvey conducted across the southern foci. Note 4 herds had a seroprevalence of >90% after the outbreak.

**Figure 8:**
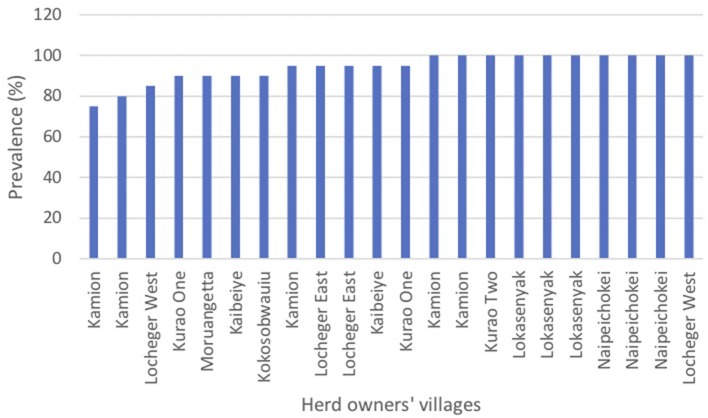
Serosurvey results from the Kamion outbreak investigation in the northern transmission focus (Kotido-Kaabong). The serosurvey following the outbreak in the Kamion & Kalapata security kraals that housed herds from numerous villages found uniformly high seroprevalences. The labels represent the home villages of the herds sampled at the security kraals. Ten of the herds sampled were 100% positive.

- Of small ruminants surveyed from the southern focus, 40.7% (285/700) were positive for PPR specific antibodies whereas 51.4% (360/700) small ruminants sampled from the northern focus tested positive for PPR specific antibodies during the serosurvey. The distribution of herd prevalence is presented for the southern and northern foci in Figures 4, 5, respectively. The spatial distribution of prevalence is presented as a map in Figure 6.
- For the outbreak investigation, 48.5% (232/478) of the sera from Loroo outbreak in southern focus and 94.3% (415/440) of the small ruminants from the northern focus outbreak (Kamion) were positive for PPR specific antibodies (Figures 7, 8, respectively). As a whole, the individual herd seroprevalences in the Loroo outbreak were greater when compared to those found in the baseline survey conducted across the hot spot before the outbreak. For example, four herds seroprevalences over 90% were detected after the outbreak.

### Sequencing

**Figure 9:**
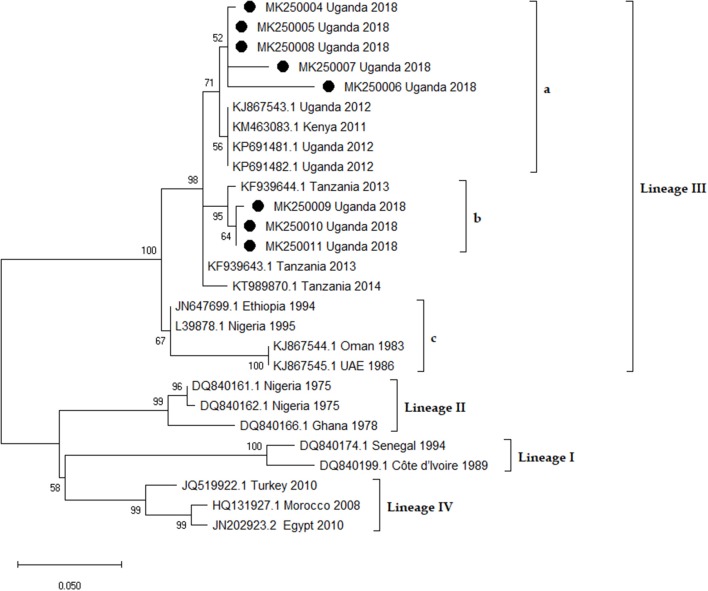
Phylogenetic analysis of Peste des petits Ruminants virus Nucleoprotein (N) gene fragment in MEGA X software. The Maximum Likelihood method and the Kimura 2-parameter [+ I] models were selected to best infer evolutionary relationship. The tree is drawn to scale, and the branch lengths correspond to the number of nucleotide substitutions per site. Bootstrap values of 1,000 replicates are indicated at each node. The rate variation model allowed for some sites to be evolutionarily invariable ([+I], 30.00% sites). This analysis involved 27 nucleotide sequences and their accession numbers and countries of origin are indicated. The sequences from this study are denoted by black circles [•] and they form two sub-clusters (a and b) in lineage III. Sub-clusters a and b are from the southern and northern foci, respectively.

**Figure 10:**
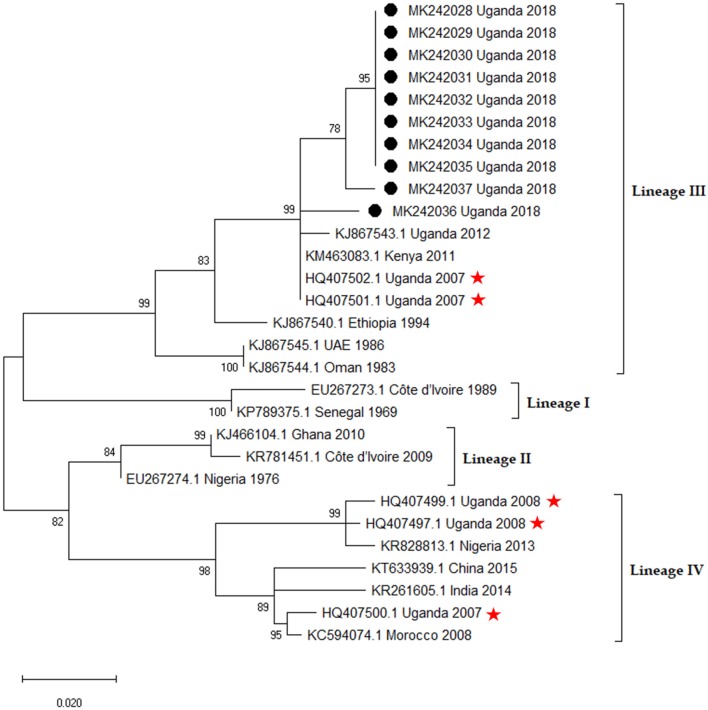
Phylogenetic analysis of Peste des petits Ruminants virus Fusion (F) gene fragment in MEGA X software. The Maximum Likelihood method employing the Kimura 2-parameter (+ I) was selected as the best model to infer evolutionary relationship. The tree is drawn to scale, and the branch lengths correspond to the number of nucleotide substitutions per site. Bootstrap values of 1,000 replicates are indicated at each node. The rate variation model allowed for some sites to be evolutionarily invariable ([+I], 36.89% sites). This analysis involved 29 nucleotide sequences and their accession numbers, country of origin and year of isolation are indicated. The sequences from this study are denoted by black circles [•] and sequences with asterisks [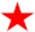] are the ones misclassified by Luka et al. (20).

Representative samples from the outbreak hotspots were randomly selected from the PCR positives and sequenced. The sequences were deposited in GenBank under accession numbers MK250004-MK2500011 and MK242028-MK2242037 for the Nucleoprotein and Fusion genes respectively. Based on the BLAST search results, representative sequences from each virus lineage including sequences from countries neighboring Uganda were retrieved from GenBank. Phylogenetic analysis, by both gene fragments revealed that sequences from this study were lineage III as shown in Figures 9, 10. Nucleoprotein gene phylogeny (Figure 9) clearly revealed two PPRV lineage III subclades (a and b) representing virus sequences from the two independent outbreaks. The northern subclade (b) was more closely related to KF939644.1 Ngorogoro than to the southern Karamoja focus. The southern focus grouped with KM 463083.1 KN5/2011, an isolate from Turkana Kenya, and KP691481.1 Uganda 2012 and KP691482.1 Uganda 2012 which originated from Kotido in 2012 in lineage III subclade a (Figure 9).

In our F gene re-analysis, previous sequences (20) formerly regarded as lineage I were classified as lineage IV whereas those regarded as lineage II clustered with lineage III in our F gene re-analysis. The F gene sequences from this study clustered with other lineage III sequences. The virus nucleotide sequences from the southern focus (Loroo) were identical or nearly so to each other but slightly different from those from the northern focus (Kamion) outbreak.

## Discussion

Most Karamojong and Pokot respondents recognized syndromes consistent with PPR and were aware of the availability of interventions to mitigate PPR. Frequently, detailed and patient dialogue on animal health issues was required to establish an accurate understanding of the experience and knowledge of the participants with respect to PPR. Practitioners of participatory epidemiology have had similar experiences in regard to PPR in Ethiopia and Tanzania (Jones et al., submitted). The complexity and diversity of language and local knowledge on PPR suggests that direct, structured questions in questionnaire surveys are unlikely to arrive at accurate understandings of community knowledge regarding PPR.

The participatory analysis identified separate northern and southern foci of PPRV transmission that were closely linked with transmission in Kenya. The presence of these transmission hotspots was supported by the surveillance exercise conducted throughout Karamoja that detected three PPR outbreaks, all located in the hotspots. Each of these virus clusters were more closely related to PPR strains of Kenyan or Tanzanian origin than they were to each other.

N gene sequences from the Kamion outbreak virus isolates were more closely related to isolates from Tanzania (Ngorongoro) as compared to those in Uganda. Ngorongoro is a northern Tanzanian district that borders Kenya and previous research advances have highlighted a belief that PPR introduction to Northern Tanzania originated from Kenya around 2008 (21). This phylogenetic finding from this study indicates that probably the PPR viruses responsible for the outbreak in Amudat district in April 2018 originated from Kenya or vice versa. More PPRV sequence data in the East African region and future studies on transboundary and cross border movement of livestock will provide more insights into the PPRV epidemiology.

The F gene sequences from Kamion outbreak (MK242036 - MK242037) were closer to isolate KJ867543, a lineage III whole genome sequence recently isolated from Uganda (22). Reanalysis of all the PPRV sequences submitted to GenBank from Uganda between 2007 and 2018, confirmed that 90% of these sequences were PPRV lineage III. However, the six F gene sequences submitted by Luka et al. (20) from Uganda retrieved from GenBank, clustered in lineage III and IV but not lineages I and II as reported by Luka et al. (20). This exact misclassification pattern of PPRV lineages in Uganda was observed and reported by the study (23). The Nucleoprotein gene sequences from this study better delineated the isolates from the two outbreak areas. This was probably so because the N gene is more polymorphic and has been credited by recent research advances as a better target for PPRV genotyping (24).

The serum sampling of the two hot spots was conducted to access population immunity level in relation to past vaccination coverage and establish a baseline herd immunity measure to support impact assessment of future vaccination programs. Small ruminant population figures for Karamoja are a subject of debate. At best, vaccine allocations do not account for more than 15–20% coverage and could not account for the level of herd immunity observed.

At the household level, a continuous range of prevalence were observed in the serosurveys from the two hot spots (Figures 4, 5). It was not possible to clearly categorize households given the nearly linear distribution of household prevalence. On the other hand, the map (Figure 6) demonstrates clustering of households in areas of high prevalence. It is not known if this represents foci of transmission or clustering of vaccination. Although the aggregate serology results indicated the presence of endemic disease, in the absence of detailed data for mapping of vaccination, serology was not useful for conclusively identifying transmission sites.

The patterns in the distribution of household seroprevalence in the two post-outbreak samples showed a clear contrast. The sample from the Loroo outbreak came from 20 separate kraals in approximately a one km radius of the index kraal. Overall, the prevalence was higher than in the random sample taken from the whole of Loroo subcounty by 7.8%. The distribution of prevalence in the herds was uniformly greater when compared to the serosurvey conducted across the southern foci. Three herds had 100% and 1 more was over 90% prevalence (Figure 7). The Kamion subcounty outbreak sample was from 22 households whose livestock were kept in two large security kraals established to guard against stock theft in raids that had recently occurred. The seroprevalence of 94.3% indicates that transmission rates were high within these large kraals (Figure 8). The pattern at Loroo with 4 kraals with 90 to 100% prevalence suggest that the between herd transmission rate was moderate, whereas the within herd transmission rate was high.

It has been argued that larger pastoral areas maintain PPR as one continuous and relatively amorphous system of transmission. The distribution and timing of reported outbreaks, in the absence of good qualitative data and genomics, appear as diffuse endemism requiring a mass response. Our findings indicate that deeper investigation of the patterns of circulation of PPR within pastoral areas using good participatory inquiry and genomics can reveal finer structure that can facilitate eradication efforts. The distribution of outbreaks identified and the cluster of strains confirmed that Karamoja has at least two systems of PPR transmission. This finding is of great significance for the targeting of eradication interventions in the global eradication of PPR.

Our findings suggest that even within the relatively small area of Karamoja, the northern and southern foci are predominately separate systems that should be explicitly addressed separately in the implementation of eradication. However, both these systems extend across the border into Kenya and any effort to address either system must be through a holistic program consisting of integrated cross-border interventions. One system is in the Pokot communities near Loroo, Uganda and Alalae, Kenya and the second in the Jie, Dodoth, Karamojong, and Turkana communities that occupy the Nakapelimoru, Loyoro, Kamion area of Uganda and the Loima area of Kenya.

The concept of targeting eradication interventions is based in an adaptive management approach. Adaptive management assumes that information is incomplete and strives to make the best decisions possible on available evidence while advocating for continued learning. The epidemiological scenario presented here is a significant step forward for the targeting of interventions, but should not be considered final nor permanent. Additional information would improve the accuracy of the scenarios and may reveal more foci or shifting relationships. Risk factors that shape transmission patterns will change over time. Participatory assessment, outbreak investigation and sampling leading to genetic analysis should be intensified within Karamoja, across Uganda and internationally.

It is proposed that the use of epidemiological analysis to target vaccination can enhance the efficacy of eradication interventions and reduce the costs of eradication. The cost of the delivery of one vaccine has been estimated as 0.30 USD (25). The resources that can be mobilized for PPR eradication are not unlimited and need to be utilized effectively in line with the program goals. Routine, institutionalized vaccination that is not targeted to viral elimination leads to suppressed endemism and is an impediment to eradication (26). Prior to vaccination, ground work to establish epidemiological goals and coordinated delivery mechanisms that achieve those goals are required.

## Data Availability

The datasets generated for this study are available on request to the corresponding author.

## Ethics Statement

Ethical approval was granted, approval numbers: IRB201701701 (University of Florida), IRB1707030 (Tufts University) and by the institutional research board of Makerere University (Research proposal SBLS/REC/17/001), University of Florida (Study #; 201701701) and Tufts University (Study #: 1707030). Institutional Animal Care and Use Committee (IACUC) was obtained from the University of Florida (Protocol #: 201709832).

## Author Contributions

JN participated in field investigations and development and optimization of laboratory analysis plan, performed spatial and phylogenetic analysis, and writing the manuscript. JC-S conducted sight assessment interviews, investigated and sampled outbreaks, facilitated risk mapping, and contributed to writing the manuscript. SO supervised development and optimization of laboratory protocols. FM supervised development and optimization of laboratory protocols. AP supported surveillance and detected, field diagnosed, and sampled the Kamion outbreak. CN participated in laboratory and phylogenetic analysis. EI facilitated focus groups, supported surveillance, and outbreak sampling. NN contributed to the design of the research and surveillance system. PN participated in field investigations. RA participated in field assessments and establishing the surveillance system. SH participated in the design of the research. JM led research and manuscript preparation and contributed to the site assessment, risk mapping, surveillance system, and outbreak investigation and sampling.

### Conflict of Interest Statement

The authors declare that the research was conducted in the absence of any commercial or financial relationships that could be construed as a potential conflict of interest.
